# One-year mortality prediction for patients with sepsis: a nomogram integrating lactic dehydrogenase and clinical characteristics

**DOI:** 10.1186/s12879-023-08636-8

**Published:** 2023-10-09

**Authors:** Jin Wang, Weiyu Fei, Qianying Song

**Affiliations:** 1 Health Management Center, The Second Hospital of Dalian Medical University, Dalian, 116023 People’s Republic of China; 2 Emergency Intensive Care Unit, The Second Hospital of Dalian Medical University, Dalian, 116023 People’s Republic of China

**Keywords:** Lactic dehydrogenase, One-year mortality, Prediction model, Sepsis

## Abstract

**Background:**

To explore the association between myocardial enzymes and one-year mortality, and establish a nomogram integrating myocardial enzymes and clinical characteristics to predict one-year mortality among sepsis patients.

**Methods:**

Data of 1,983 sepsis patients were extracted from Medical Information Mart for Intensive Care III database in this retrospective cohort study. All participants were randomly split into the training set for the development of model and testing set for the internal validation at the ratio of 7:3. Univariate logistic regression was used to screen variables with statistical differences which were made for stepwise regression, obtaining the predictors associated with one-year mortality of sepsis patients. Adopted multivariate logistic regression to assess the relationship between myocardial enzymes and one-year mortality of sepsis patients. A nomogram was established in predicting the one-year survival status of sepsis patients, and the performance of developed model were compared with LDH alone, sequential organ failure assessment (SOFA), simplified acute physiology score II (SAPS II) by receiver operator characteristic, calibration, and decision curves analysis.

**Results:**

The result found that LDH was associated with one-year mortality of sepsis patients [odds ratio = 1.28, 95% confidence interval (CI): 1.18–1.52]. Independent predictors, including age, gender, ethnicity, potassium, calcium, albumin, hemoglobin, alkaline phosphatase, vasopressor, Elixhauser score, respiratory failure, and LDH were identified and used to establish the nomogram (LDH-model) for predicting one-year mortality for sepsis patients. The predicted performance [area under curve (AUC) = 0.773, 95%CI: 0.748–0.798] of this developed nomogram in the training and testing sets (AUC = 0.750, 95%CI: 0.711–0.789), which was superior to that of LDH alone, SOFA score, SAPS II score. Additionally, calibration curve indicated that LDH-model may have a good agreement between the predictive and actual outcomes, while decision curve analysis demonstrated clinical utility of the LDH-model.

**Conclusion:**

LDH level was related to the risk of one-year mortality in sepsis patients. A prediction model based on LDH and clinical features was developed to predict one-year mortality risk of sepsis patients, surpassing the predictive ability of LDH alone as well as conventional SAPS II and SOFA scoring systems.

**Supplementary Information:**

The online version contains supplementary material available at 10.1186/s12879-023-08636-8.

## Background

Sepsis, as a kind of complex disorder, is characterized by severe and potentially lethal infection accompanied by dysfunction of vital organs [1, 2]. At present, the incidence and mortality of sepsis remains high, which was considered as one of the leading causes of death worldwide [3]. The annual incidence of sepsis in the United States was estimated to exceed 1.5 million cases, resulting in approximately 250,000 fatalities [4]. Previous studies have indicated a high incidence of myocardial organic damage in the early stage of sepsis, rendering these patients susceptible to heart failure and arrhythmia, thereby contributing to a poor prognosis among septic patients [5–7]. The high mortality and morbidity of sepsis impose a substantial financial burden on the healthcare system [8]. Therefore, it is crucial to identify the prognostic factors associated with sepsis aimed to provide accurate treatment and increase the chances of survival.

The myocardial injury has been widely recognized as the main manifestation of multiple organ dysfunction in sepsis [9], and it has been observed that sepsis may cause myocardial damage. Myocardial enzymes are important indicators in assessing both myocardial function and myocardial damage. In general, myocardial enzymes included creatine kinase (CK), creatine kinase isoenzyme (CK-MB), aspartic transoxygenase (AST), and lactic dehydrogenase (LDH). The levels of myocardial enzymes could reflect the extent of cellular damages and vary with the severity of infection [10]. The levels of LDH have also been observed to increase in accordance with the severity of the infection, suggesting that LDH levels may serve as a prognostic factor for sepsis [11]. In addition, Jeon, et al. have reported that LDH to albumin (LDH/ALB) ratio might be a prognostic factor among patients with severe infection requiring intensive care [10]. Clinical decision tools could help clinician to identify those at risk of poor outcomes for sepsis patients. Nowadays, several studies have developed prediction models to predict the prognosis of sepsis patients, including machine learning model, Sequential Organ Failure Assessment (SOFA) score, Quick SOFA (qSOFA) score, and Logistic Organ Dysfunction System (LODS) score [12, 13]. Nevertheless, to the best of our knowledge, there were few studies to combine clinical features to make a diagnosis to date.

Herein, the purpose of this study was to investigate the relationship between various myocardial enzymes and one-year mortality among patients with sepsis using the Medical Information Mart for Intensive Care (MIMIC-III) database, and to establish a prediction model in which combining clinical features in predicting the risk of one-year mortality in patients with sepsis.

## Methods

### Data sources and study population

All information of this study were obtained from MIMIC-III database, which is a large, single-center, freely available database [14]. The database contained comprehensive clinical data of patients admitted to the Beth Israel Deaconess Medical Center in Boston, Massachusetts between 2001–2012, and was released on 2016. Over 40,000 patients who have been exempted from personal information were included in this database [15].

**Fig. 1:**
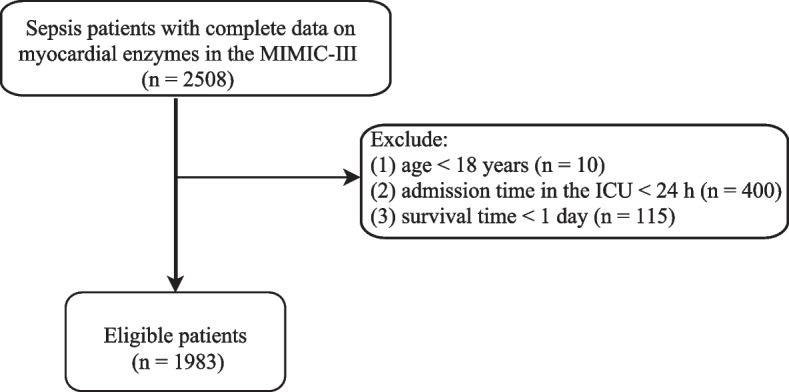
Flowchart showing the selection of study population. (ICU, medical intensive care unit)

We selected patients from MIMIC-III database between 2001–2012 in this retrospective cohort study. We analyzed only septic patients admitted to the ICU for the first time. The inclusion criteria: (1) patients were diagnosed with sepsis was defined as life-threatening organ dysfunction caused by a dysregulated host response to infection according to the Sepsis-3 criteria [16, 17]; (2) patients had complete data on myocardial enzymes. The exclusion criteria are as follows: (1) patients aged < 18 years; (2) admission time < 24 h in the ICU; (3) survival time < 1 day (Fig. 1). The experimental protocol was approved by the Institutional Review Boards of Beth Israel Deaconess Medical Center (Boston, MA) and the Massachusetts Institute of Technology (Cambridge, MA). This study did not require an approval of the Second Hospital of Dalian Medical University ethics committee because the data about included patients derived from publicly available database. All individuals provided written informed consent before participating in the study. All methods were carried out in accordance with relevant guidelines and regulations (declaration of Helsinki).

### Potential predictors

The demographic data about eligible patients were extracted from the MIMIC-III database: age (years), gender, ethnicity, marital status. The vital signs and laboratory values: respiratory rate (times/min), temperature (℃), heart rate (times/min), systolic blood pressure (SBP, mmHg), diastolic blood pressure (DBP, mmHg), mean arterial pressure (MAP, mmHg), pulse oxygen saturation (SPO_2_), white blood cell count (WBC, K/uL), red blood count (RBC, m/uL), sodium (mEq/L), potassium (mEq/L), phosphate (mg/dL), calcium (mg/dL), magnesium (mg/dL), platelet count (PLT, K/uL), pH, lactate (mmol/L), glucose (mg/dL), creatinine (mg/dL), blood urea nitrogen (BUN, mg/dL), bicarbonate (mEq/L), albumin, total bilirubin (TBIL, mg/dL), hematocrit (%), hemoglobin (g/dL), mean corpuscular hemoglobin concentration (MCHC), alkaline phosphatase (ALP), alanine aminotransferase (ALT, U/L), LDH (U/L), CK (U/L), CK-MB (U/L), AST (U/L), oxygen partial pressure (PO_2_), partial pressure of carbon dioxide (PCO_2_). Comorbidities: congestive heart failure, malignant tumor, atrial fibrillation, respiratory failure, septic shock and Elixhauser score. Severity score: sequential organ failure assessment (SOFA) score and simplified acute physiology score II (SAPS II). Outcomes data: intensive care unit (ICU) type, length of stay, renal replacement therapy (RRT), ventilation and vasopressor. All variables were collected within 24 h of patients’ admission to the ICU. Length of stay was considered as the admission time in the ICU.

To describe comorbidities more succinctly, we used Elixhauser score. Elixhauser score includes 30 comorbidities, such as hypertension, diabetes, renal failure, hypothyroidism, cardiac arrhythmias and so on [18, 19].

### Outcomes and follow-up

The outcome was one-year survival status of sepsis patients after ICU admission. Follow-up started at the time of patients' admission, and was terminated when death occurred. The follow-up duration was one-year and the median follow-up time was 143.19 (15.45, 365.00) days.

### Statistical analysis

Kolmogorov–Smirnov was used to test the normality of measurement data. Mean ± standard deviation (Mean ± SD) and Median and quartile spacing [M (Q1, Q3)] described the normal and non-normal distribution of the measurement data, respectively; and the comparison between the two groups was performed by Student’s t-test and rank-sum test, respectively. The categorical data was depicted by the number of cases and composition ratio n (%), and difference of groups were compared by χ^2^ test. These missing values were interpolated by using multiple interpolation method, and sensitivity analysis was performed by using data filled before and after between groups (Supplemental Table 1).

First, the total participants were randomly split into the training set for the development of model and the testing set for the internal validation of model at the ratio of 7:3, and we conducted a balance test between two sets. Then, the patients in the training set were subsequently categorized into two groups, namely the death group and the survival group, based on their one-year mortality or survival status. Subsequently, a comparison of variables was conducted between these two groups. We utilized univariate logistic regression to identify variables exhibiting statistically significant differences, and made these variables to stepwise regression to obtain predictors associated with one-year mortality of sepsis patients. A multivariate logistic regression analysis was introduced to assess the relationship between various myocardial enzymes and one-year mortality among patients with sepsis: Model 1 was a coarse model; Model 2 adjusted age, gender, ethnicity, potassium, calcium, albumin, hemoglobin, ALP, vasopressor, Elixhauser score, and respiratory failure. Subsequently, a prediction model combining myocardial enzymes and other predictors was established in predicting the one-year survival status of patients with sepsis. The performance of developed model was compared with SOFA score, SAPSII score by some indexes, including receiver operator characteristic curve (ROC) analysis, calibration curve and decision curve analysis (DCA). The inter-group comparisons were performed using SAS 9.4 software, and all statistics were performed using R 3.6.3 software. All statistical tests were conducted by two-sided tests, and *P* < 0.05 was considered as statistically significant.

## Results

### Participants’ characteristics

We excluded some patients who were aged < 18 years (*n* = 10), were < 24 h of admission time in the ICU (*n* = 400) and were < 1 day of survival time (*n* = 115). A total of 1,983 eligible patients were eventually included, which were randomly divided into training set (*n* = 1,388) and testing set (*n* = 595). The baseline characteristics for all enrolled patients were listed in Supplemental Table 2. The mean age of the total population was 67.79 ± 15.31 years old. 46.44% of sepsis patients had congestive heart failure, 26.42% had a history of malignant tumor, and 42.16% had atrial fibrillation. The median Elixhauser score was 24.00 for total population. The one-year mortality rate of sepsis patients reached 56.13% in this study. Additionally, the differences of all variables between the training and testing sets were not statistically significant (*P* > 0.05), indicating that the data from two groups were well-balanced and comparable. As shown in Table 1, patients in the training set were classified into death group (*n* = 788) and survival group (*n* = 600) based on one-year survival status. We observed that some underlying diseases were significantly different between the two groups, including congestive heart failure, malignant tumor, atrial fibrillation, respiratory failure, and Elixhauser score. The number of sepsis patients with congestive heart failure, malignant tumor, atrial fibrillation, and respiratory failure was higher in the death group compared to the survival group.

**Table 1:** The patients’ characteristics of survival group and death group in the training set

Variables	Total (*n* = 1388)	Survival group (*n* = 668)	Death group (*n* = 824)	Statistics	*P*
Age, years, Mean ± SD	68.02 ± 15.47	64.06 ± 16.12	71.04 ± 14.24	t = -8.40	< 0.001
Gender, n (%)				χ^2^ = 4.202	0.040
Female	586 (42.22)	272 (45.33)	314 (39.85)		
Male	802 (57.78)	328 (54.67)	474 (60.15)		
Marital status, n (%)				χ^2^ = 0.458	0.499
Married	668 (48.13)	295 (49.17)	373 (47.34)		
Not married	720 (51.87)	305 (50.83)	415 (52.66)		
Ethnicity, n (%)				χ^2^ = 14.338	0.002
White	1128 (81.27)	464 (77.33)	664 (84.26)		
Asian	43 (3.10)	28 (4.67)	15 (1.90)		
Black	142 (10.23)	72 (12.00)	70 (8.88)		
Hispanic	75 (5.40)	36 (6.00)	39 (4.95)		
ICU type, n (%)				χ^2^ = 5.795	0.215
CCU	140 (10.09)	53 (8.83)	87 (11.04)		
CSRU	56 (4.03)	18 (3.00)	38 (4.82)		
MICU	937 (67.51)	421 (70.17)	516 (65.48)		
SICU	176 (12.68)	76 (12.67)	100 (12.69)		
TSICU	79 (5.69)	32 (5.33)	47 (5.96)		
Length of stay, days, M (Q_1_, Q_3_)	5.67 (2.81, 12.73)	5.02 (2.59, 12.17)	6.07 (2.90, 12.80)	Z = -1.573	0.116
Respiratory rate, times/min, Mean ± SD	21.53 ± 6.62	21.56 ± 6.64	21.51 ± 6.60	t = 0.15	0.881
Temperature, ℃, Mean ± SD	36.72 ± 2.50	36.88 ± 3.54	36.59 ± 1.22	t = 1.90	0.058
Heart rate, times/min, Mean ± SD	97.36 ± 21.79	98.76 ± 21.32	96.29 ± 22.09	t = 2.09	0.037
SBP, mmHg, Mean ± SD	115.05 ± 25.39	115.60 ± 24.79	114.63 ± 25.84	t = 0.71	0.479
DBP, mmHg, Mean ± SD	61.62 ± 18.60	63.55 ± 19.27	60.15 ± 17.94	t = 3.39	< 0.001
MAP, mmHg, Mean ± SD	76.36 ± 19.21	77.16 ± 19.75	75.76 ± 18.78	t = 1.34	0.180
SPO_2_, Mean ± SD	95.82 ± 7.35	95.98 ± 7.30	95.70 ± 7.40	t = 0.71	0.478
WBC, K/uL, M (Q_1_, Q_3_)	11.90 (7.70, 17.40)	12.50 (8.20, 18.10)	11.70 (7.40, 16.80)	Z = 1.925	0.054
RBC, m/uL, Mean ± SD	3.75 ± 0.77	3.87 ± 0.76	3.66 ± 0.77	t = 5.26	< 0.001
Sodium, mEq/L, Mean ± SD	137.59 ± 6.55	137.64 ± 6.45	137.54 ± 6.62	t = 0.29	0.772
Potassium, mEq/L, Mean ± SD	4.43 ± 1.00	4.26 ± 0.95	4.56 ± 1.02	t = -5.57	< .001
Phosphate, mg/dL, M (Q_1_, Q_3_)	3.60 (2.80, 4.60)	3.30 (2.60, 4.30)	3.80 (3.00, 4.90)	Z = -6.595	< 0.001
Calcium, mg/dL, Mean ± SD	8.21 ± 1.06	8.08 ± 1.05	8.31 ± 1.06	t = -4.03	< 0.001
PLT, K/uL, M (Q_1_, Q_3_)	210.00 (133.00, 303.00)	215.00 (145.50, 292.00)	207.50 (123.00, 311.00)	Z = 1.093	0.274
pH, Mean ± SD	7.34 ± 0.12	7.33 ± 0.12	7.34 ± 0.12	t = -1.31	0.191
Lactate, mmol/L, M (Q_1_, Q_3_)	2.30 (1.50, 3.80)	2.20 (1.50, 3.80)	2.30 (1.50, 3.70)	Z = -0.597	0.550
Magnesium, mg/dL, Mean ± SD	1.94 ± 0.51	1.87 ± 0.48	2.00 ± 0.52	t = -4.75	< 0.001
Glucose, mg/dL, M (Q_1_, Q_3_)	132.00 (106.00, 177.00)	134.00 (108.00, 182.50)	131.00 (105.00, 174.00)	Z = 1.747	0.081
Creatinine, mg/dL, M (Q_1_, Q_3_)	1.50 (1.00, 2.50)	1.40 (1.00, 2.25)	1.60 (1.00, 2.70)	Z = -2.998	0.003
BUN, mg/dL, M (Q_1_, Q_3_)	31.50 (20.00, 51.00)	27.50 (18.00, 44.00)	35.00 (22.00, 57.00)	Z = -5.852	< 0.001
Bicarbonate, mEq/L, Mean ± SD	22.56 ± 5.56	22.28 ± 5.28	22.78 ± 5.75	t = -1.68	0.093
Albumin, Mean ± SD	2.90 ± 0.65	2.97 ± 0.67	2.85 ± 0.62	t = 3.37	< 0.001
TBIL, mg/dL, M (Q_1_, Q_3_)	0.70 (0.40, 1.50)	0.60 (0.40, 1.30)	0.70 (0.40, 1.70)	Z = -2.038	0.042
Hematocrit, %, Mean ± SD	33.99 ± 6.51	34.94 ± 6.50	33.26 ± 6.43	t = 4.78	< 0.001
Hemoglobin, g/dL, Mean ± SD	11.25 ± 2.21	11.64 ± 2.22	10.96 ± 2.16	t = 5.72	< 0.001
MCHC, Mean ± SD	33.12 ± 1.66	33.31 ± 1.67	32.97 ± 1.64	t = 3.73	< 0.001
ALP, M (Q_1_, Q_3_)	99.00 (70.00, 155.00)	91.00 (65.00, 135.00)	103.00 (73.00, 168.00)	Z = -4.354	< 0.001
ALT, U/L, M (Q_1_, Q_3_)	30.00 (17.00, 64.00)	30.50 (18.00, 64.10)	30.00 (17.00, 64.00)	Z = 0.373	0.709
AST, U/L, M (Q_1_, Q_3_)	43.00 (25.00, 100.00)	43.00 (25.00, 86.00)	43.00 (26.00, 110.00)	Z = -0.878	0.380
LDH, U/L, M (Q_1_, Q_3_)	281.00 (213.00, 426.50)	258.50 (206.00, 371.00)	300.00 (219.00, 486.50)	Z = -4.829	< 0.001
CK, U/L, M (Q_1_, Q_3_)	113.00 (47.00, 285.00)	134.50 (60.00, 329.50)	97.50 (40.00, 259.00)	Z = 4.279	< 0.001
CK-MB, U/L, M (Q_1_, Q_3_)	4.00 (3.00, 8.00)	4.00 (2.00, 8.00)	4.61 (3.00, 8.50)	Z = -2.308	0.021
PO_2_, M (Q_1_, Q_3_)	98.00 (68.00, 178.20)	100.00 (69.00, 184.50)	95.20 (67.00, 174.50)	Z = 0.605	0.545
PCO_2_, M (Q_1_, Q_3_)	39.00 (33.00, 47.00)	39.30 (34.00, 47.00)	39.00 (33.00, 47.00)	Z = 0.909	0.363
Congestive heart failure, n (%)				χ^2^ = 4.584	0.032
No	727 (52.38)	334 (55.67)	393 (49.87)		
Yes	661 (47.62)	266 (44.33)	395 (50.13)		
Malignant tumor, n (%)				χ^2^ = 12.904	< 0.001
No	1022 (73.63)	471 (78.50)	551 (69.92)		
Yes	366 (26.37)	129 (21.50)	237 (30.08)		
Atrial fibrillation, n (%)				χ^2^ = 13.799	< 0.001
No	796 (57.35)	378 (63.00)	418 (53.05)		
Yes	592 (42.65)	222 (37.00)	370 (46.95)		
Respiratory failure, n (%)				χ^2^ = 20.654	< 0.001
No	607 (43.73)	304 (50.67)	303 (38.45)		
Yes	781 (56.27)	296 (49.33)	485 (61.55)		
Septic shock, n (%)				χ^2^ = 1.338	0.247
No	637 (45.89)	286 (47.67)	351 (44.54)		
Yes	751 (54.11)	314 (52.33)	437 (55.46)		
Elixhauser score, M (Q_1_, Q_3_)	24.00 (14.00, 34.00)	18.00 (9.00, 29.00)	27.00 (18.00, 36.00)	Z = -10.266	< 0.001
SOFA total score, M (Q_1_, Q_3_)	7.00 (5.00, 10.00)	7.00 (4.00, 10.00)	8.00 (5.00, 11.00)	Z = -5.016	< 0.001
SAPS II, M (Q_1_, Q_3_)	47.00 (37.00, 57.00)	41.00 (33.00, 51.00)	50.00 (41.00, 61.00)	Z = -11.426	< 0.001
RRT, n (%)				χ^2^ = 7.715	0.005
No	1243 (89.55)	553 (92.17)	690 (87.56)		
Yes	145 (10.45)	47 (7.83)	98 (12.44)		
Ventilation, n (%)				χ^2^ = 11.529	< 0.001
No	457 (32.93)	227 (37.83)	230 (29.19)		
Yes	931 (67.07)	373 (62.17)	558 (70.81)		
Vasopressor, n (%)				χ^2^ = 35.158	< 0.001
No	1040 (74.93)	497 (82.83)	543 (68.91)		
Yes	348 (25.07)	103 (17.17)	245 (31.09)		
Follow-up time, M (Q_1_, Q_3_)	137.96 (15.05, 365.00)	365.00 (365.00, 365.00)	19.27 (7.24, 51.43)	Z = 33.330	< 0.001

*ICU* Intensive care unit, *CCU* Coronary care unit, *CSRU* Cardiac surgery recovery unit, *MICU* Medical intensive care unit, *SICU* Surgical intensive care unit, *TSICU* Trauma/surgical intensive care unit, *SBP* Systolic blood pressure, *DBP* Diastolic blood pressure, *MAP* Mean arterial pressure, *SPO*_*2*_ Pulse oxygen saturation, *WBC* White blood cell count, *RBC* Red blood count, *PLT* Platelet count, *BUN* Blood urea nitrogen, *TBIL* Total bilirubin, *MCHC* Mean corpuscular hemoglobin concentration, *ALP* Alkaline phosphatase, *ALT* Alanine aminotransferase, *CK* Creatine kinase, *CK-MB* Creatine kinase isoenzyme, *AST* Aspartic transoxygenase, *LDH* Lactic dehydrogenase, *PO*_*2*_ Oxygen partial pressure, *PCO*_*2*_ Partial pressure of carbon dioxide, *SOFA* Sequential organ failure assessment, *SAPS* Simplified acute physiology score, *RRT* Renal replacement therapy

### The association of different myocardial enzymes and one-year mortality among patients with sepsis

We performed a univariate logistic regression analysis, and the result showed that age, gender, ethnicity, potassium, calcium, albumin, hemoglobin, ALP, vasopressor, Elixhauser score and respiratory failure were associated with one-year mortality of sepsis patients, which were possible predictors (Supplemental Table 3).

Table 2 displays the relationship between different myocardial enzymes and one-year mortality among patients with sepsis. The result indicated that LDH was associated with the risk of one-year mortality among sepsis patients [Model 1: odds ratio (OR) = 1.28, 95% confidence interval (CI): 1.09–1.49, *P* = 0.002], with an adjusted OR of 1.28 (Model 2, 95% CI: 1.18–1.52, *P* = 0.005). Furthermore, we also found that there were no statistically significant differences between the AST, CK, CK-MB and one-year mortality for sepsis patients.

**Table 2:** The association of different myocardial enzymes and one-year mortality among patients with sepsis

Variables	Model 1			Model 2		
	**OR**	**95%CI**	***P***	**OR**	**95%CI**	***P***
LDH	1.28	1.09–1.49	0.002	1.28	1.08–1.52	0.005
AST	1.06	0.94–1.20	0.332	1.05	0.89–1.23	0.551
CK-MB	1.04	0.93–1.16	0.508	1.06	0.94–1.21	0.349
CK	0.94	0.82–1.08	0.404	0.98	0.87–1.11	0.795

Model 1, crude model; Model 2, adjusted age, gender, ethnicity, potassium, calcium, albumin, hemoglobin, alkaline phosphatase, vasopressor, Elixhauser score and respiratory failure

*CK* Creatine kinase, *CK-MB* Creatine kinase isoenzyme, *AST* Aspartic transoxygenase, *LDH* Lactic dehydrogenase, *OR* Odds ratio, *CI* Confidence interval

### Development of prediction model (LDH-model)

**Fig. 2:**
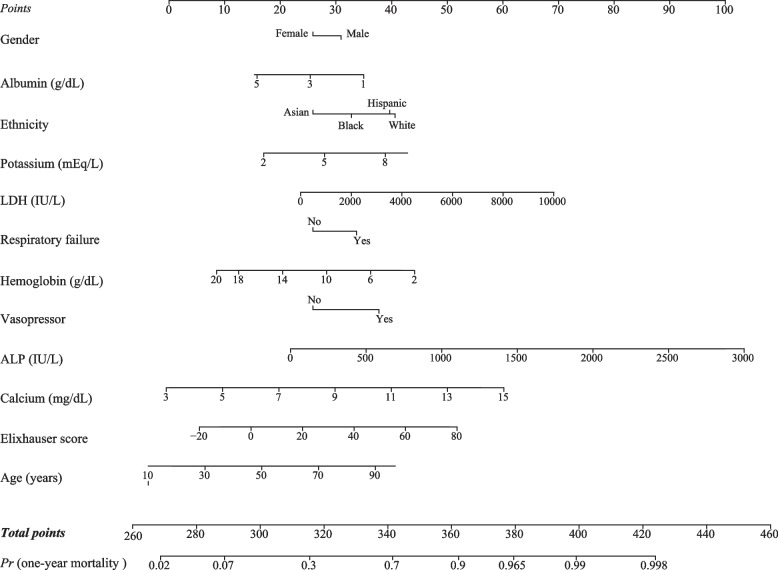
A nomogram in predicting the probability of the one-year mortality of patients with sepsis. (LDH, lactic dehydrogenase; ALP, alkaline phosphatase)

A prediction model (LDH-model) integrating LDH, age, gender, ethnicity, potassium, calcium, albumin, hemoglobin, ALP, vasopressor, Elixhauser score, and respiratory failure was established in the training set. In order to visualize the developed LDH-model, a nomogram was plotted to predict the probability of the one-year mortality of patients with sepsis (Fig. 2). An online prediction nomogram: https://songqy.shinyapps.io/DynNomapp/

### Sample

**Fig. 3:**
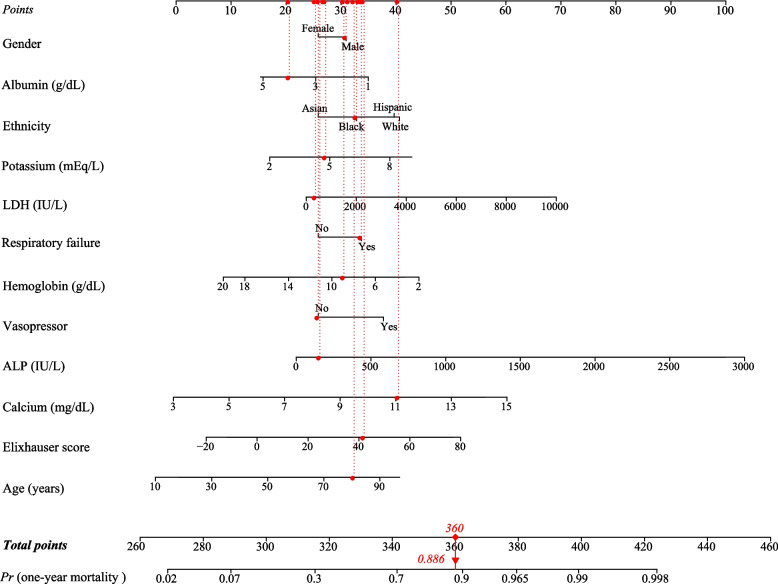
An example for the application of the nomogram. (LDH, lactic dehydrogenase; ALP, alkaline phosphatase)

We randomly chose a male patient with sepsis who age was 81 years old, LDH was 371 IU/L, potassium was 4.8 mEq/L, calcium was 11.1 mg/dL, albumin was 4 g/dL, hemoglobin was 8.9 g/dL, ALP was 159 IU/L, had not used vasopressor, Elixhauser score was 42, and had respiratory failure. The total point was 360, and the predicted probability of one-year mortality was 0.886 (Fig. 3), which suggested a higher risk of one-year mortality and was consistent with the actual outcome of the patient.

### Validation and comparison of prediction model

**Fig. 4:**
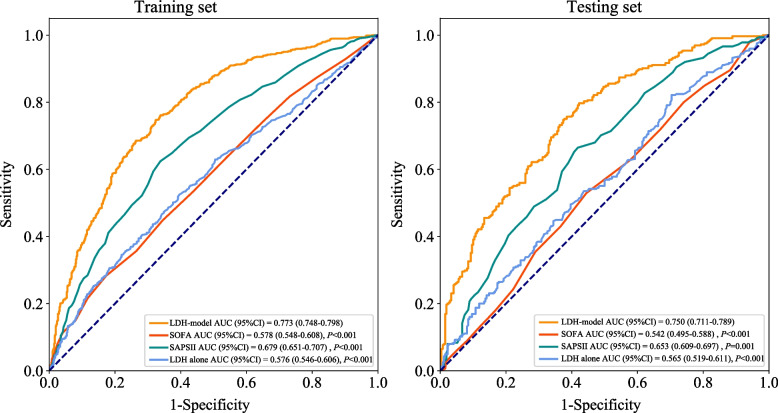
Receiver operating characteristic curves of prediction models in the training and testing sets. (AUC, area under the curve; CI, confidence interval; LDH, lactic dehydrogenase; SOFA, sequential organ failure assessment; SAPS, simplified acute physiology score)

In order to verify the performance of the established LDH-model, which combines LDH and clinical features, we adopted data from the testing set for internal validation. According to the ROC analysis, the AUC of LDH-model was 0.773 (95%CI: 0.748–0.798) with the sensitivity of 0.761 (95%CI: 0.732–0.791), the specificity of 0.662 (95%CI: 0.624–0.700), and the accuracy of 0.718 (95%CI: 0.695–0.742) in the training set, and the AUC was 0.750 (95%CI: 0.711–0.789) with the sensitivity of 0.751 (95%CI: 0.704–0.798), the specificity of 0.619 (95%CI: 0.561–0.676), and the accuracy of 0.691 (95%CI: 0.654–0.728) in the testing set (Table 3, Fig. 4). Simultaneously, Table 3 also revealed that LDH-model exhibited superior predictive performance compared to LDH alone, SOFA and SAPS II scores in both datasets.

**Table 3:** Comparison of models in predicting the one-year mortality of sepsis patients

Models	Sets	Cut off	Sensitivity (95%CI)	Specificity (95%CI)	PPV (95%CI)	NPV (95%CI)	AUC (95%CI)	Accuracy (95%CI)
LDH-model	Training set	0.530	0.761 (0.732–0.791)	0.662 (0.624–0.700)	0.747 (0.717–0.777)	0.679 (0.641–0.716)	0.773 (0.748–0.798)	0.718 (0.695–0.742)
	Testing set		0.751 (0.704–0.798)	0.619 (0.561–0.676)	0.703 (0.655–0.751)	0.673 (0.615–0.732)	0.750 (0.711–0.789)	0.691 (0.654–0.728)
SOFA	Training set	0.629	0.283 (0.252–0.314)	0.827 (0.796–0.857)	0.682 (0.631–0.732)	0.467 (0.437–0.498)	0.578 (0.548–0.608)	0.518 (0.492–0.544)
	Testing set		0.178 (0.137–0.220)	0.833 (0.789–0.878)	0.563 (0.467–0.659)	0.457 (0.413–0.501)	0.542 (0.495–0.588)	0.476 (0.436–0.516)
SAPS II	Training set	0.570	0.624 (0.591–0.658)	0.662 (0.624–0.700)	0.708 (0.674–0.742)	0.573 (0.536–0.610)	0.679 (0.651–0.707)	0.640 (0.615–0.666)
	Testing set		0.594 (0.540–0.647)	0.630 (0.572–0.687)	0.659 (0.604–0.713)	0.563 (0.507–0.619)	0.653 (0.609–0.697)	0.610 (0.571–0.649)
LDH alone	Training set	0.556	0.525 (0.491–0.560)	0.603 (0.564–0.642)	0.635 (0.598–0.672)	0.492 (0.456–0.528)	0.576 (0.546–0.606)	0.559 (0.533–0.585)
	Testing set		0.542 (0.487–0.596)	0.530 (0.470–0.589)	0.581 (0.525–0.636)	0.490 (0.432–0.547)	0.565 (0.519–0.611)	0.536 (0.496–0.576)

LDH-model: a predicting model integrating LDH and clinical features (including age, gender, ethnicity, potassium, calcium, albumin, hemoglobin, alkaline phosphatase, vasopressor, Elixhauser score, and respiratory failure)

*LDH* Lactic dehydrogenase, *SOFA* Sequential organ failure assessment, *SAPS* Simplified acute physiology score, *PPV* Positive predictive value, *NPV* Negative predictive value, *AUC* Area under the curve, *CI* Confidence interval

**Fig. 5:**
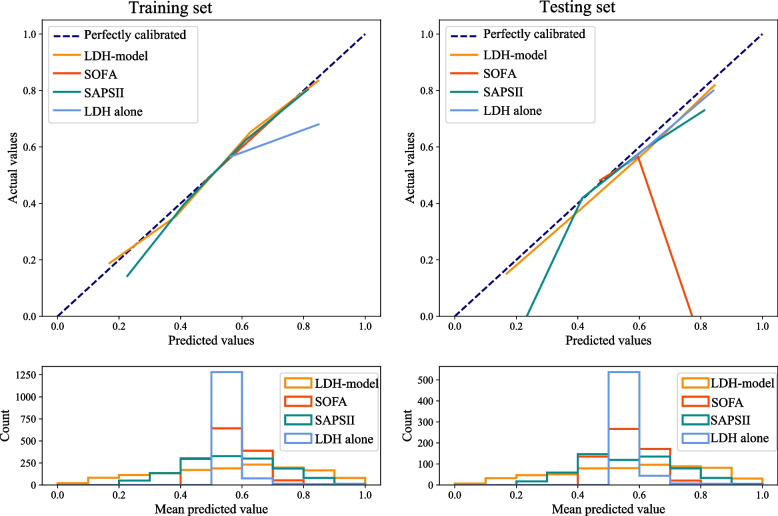
Calibration curves of prediction models in the training and testing sets. (LDH, lactic dehydrogenase; SOFA, sequential organ failure assessment; SAPS, simplified acute physiology score)

**Fig. 6:**
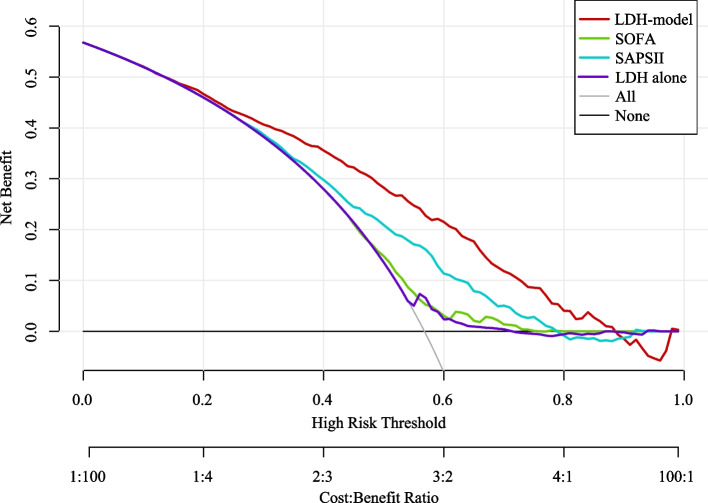
Decision curve analysis of prediction models. (LDH, lactic dehydrogenase; SOFA, sequential organ failure assessment; SAPS, simplified acute physiology score)

Not only that, the calibration curve was used to describe the fitting ability of the LDH-model in the training and testing sets. Figure 5 exhibits that the established LDH-model has a better agreement between the predictive and actual outcomes than LDH alone, SOFA score, and SAPS II score. Additionally, we also plotted the DCA curves to compare clinical application of this developed LDH-model and LDH alone, SOFA score, SAPS II score (Fig. 6). It could be found from the DCA curves that under the same risk, the benefits of LDH-model were higher than LDH alone, SOFA score, SAPS II score, which indicating that the established LDH-model has a higher clinical value than other scores.

## Discussion

In this retrospective cohort study, we assessed the relationship between various myocardial enzymes and risk of one-year mortality among sepsis patients. The findings showed that LDH was associated with the risk of one-year mortality of sepsis patients. Importantly, we developed a predicting model integrating LDH and clinical features (including age, gender, ethnicity, potassium, calcium, albumin, hemoglobin, ALP, vasopressor, Elixhauser score and respiratory failure) and draw a nomogram, predicting the risk of one-year mortality for patients with sepsis. The established LDH-model might have a good performance by internally validated compared with LDH alone, SOFA score, SAPS II score.

LDH is widely distributed in various tissues and cells, serving as a diagnostic marker for diseases and tissue damage [20]. Several studies have reported that increased LDH level was associated with the higher risk of severity and mortality of some diseases, such as coronavirus disease 2019, critically ill patients with acute kidney injury, postoperative pneumonia in patients with aneurysmal subarachnoid hemorrhage so on [21–23], and has been assessed as a prognostic factor for many diseases. In general, the progression of the infection may cause some changes in the levels of several biomarkers. Lu, et al., pointed out that serum LDH was probably associated with 28-day mortality among patients with sepsis [11]. In our study, LDH level was found to be associated with the risk of the one-year mortality among patients with sepsis. The LDH facilitates the conversion of pyruvate into lactic acid. As a metabolite of aerobic glycolysis, large amounts of lactic acid accumulate inflammatory mediators and lactate during glucose metabolic reprogramming, which may potentially contribute to increased mortality in patients with sepsis [11].

Nowadays, several conventional prognostic scoring systems (including SAPS II and SOFA) and prediction model have been developed to predict the mortality risk of patients with sepsis [24]. In the study of Hu et al., they evaluated the value of SAPS II and SOFA scoring systems on predicting ICU mortality in patients with sepsis, and the result found that the SOFA and SAPS II scoring systems could predict ICU mortality in patients with sepsis, but SAPSII scores had a better predictive value than SOFA scores [25]. Hou and colleagues developed a machine learning approach using XGboost to predict the 30-days mortality for MIMIC-III patients with sepsis-3. Simultaneously, they also pointed out that the specificity and sensitivity of SAPS II scoring systems were relatively low, and the predictive performance was worse than multivariate predictive models [14]. In this retrospective cohort study, we explored the association of myocardial enzymes and the prognosis of sepsis. An interesting study is that we developed a prediction model integrating LDH and age, gender, ethnicity, potassium, calcium, albumin, hemoglobin, ALP, vasopressor, Elixhauser score and respiratory failure to predict the risk of one-year mortality for sepsis patients. The ROC, calibration and DCA curves have demonstrated the good performance of the developed LDH-model compared with traditional SAPS II, SOFA scoring systems and LDH alone in one-year prediction of probability of septic mortality. The proposed LDH-model might assist clinicians to further know the prognosis of patients suffering from sepsis, and customize precise management and treatment, which will be crucial to improve the survival chances of patients.

The strength of this study was mainly as follows: this study included a relatively large sample size, which supported the credibility of the conclusion. In addition, it is the first time to predict one-year mortality of patients with sepsis by the prediction model integrating LDH and clinical features, and compared to traditional scoring system and LDH alone, and meanwhile verified by nomogram, ROC curves, calibration curves and DCA curves. Our study had several limitations. Firstly, because of all data was collected from MIMIC-III database, and most of sepsis patients were white, which might cause a potential bias. Secondly, we excluded some patients who had missing data on myocardial enzymes. These patients might affect the representativeness of the sample, and there may be an introduce bias. More prospective studies with larger sample sizes are still needed in the future. Thirdly, because the population used in this study was derived from MIMIC-III database, we could not know the interval time between data collection and sepsis diagnosis. In addition, we did not an external validation about the established LDH-model. Despite these limitations, the prediction model integrating LDH and clinical features also may have an acceptable accuracy in predicting one-year mortality risk in patients with sepsis.

## Conclusion

LDH level was associated with one-year mortality risk among patients with sepsis. We developed a nomogram combining LDH and clinical characteristics (LDH-model) to predict one-year mortality risk of sepsis patients, surpassing the predictive ability of LDH alone as well as conventional SAPS II and SOFA scoring systems. This developed nomogram may assist clinicians to further know the prognosis of patients suffering from sepsis, and customize precise management and treatment, which will be crucial to improve the survival chances of patients. A large external cohort would be still needed to further enhance the effectiveness and credibility of our model in future studies. In addition, the developed nomogram might be widely applied in clinical practice to facilitate medical decision-making. However, future research could also focus on simplifying the LDH-model and further expanding its applicability, such as integrating it into mobile devices or computer applications.

### Supplementary Information

**Additional file 1: Supplemental Table 1. **Sensitivity analysis of missing data before and after interpolation. **Supplemental Table 2.** The characteristics of patients in training set and testing set. **Supplemental Table 3.** The screening of predictors.

## Data Availability

The Datasets are available from the MIMIC-III database, https://physionet.org/content/mimiciii/1.4/.

## Abbreviations

CK: Creatine kinase
CK-MB: Creatine kinase isoenzyme
AST: Aspartic transoxygenase
LDH: Lactic dehydrogenase
LDH/ALB: LDH to albumin
SOFA: Sequential Organ Failure Assessment
qSOFA: Quick SOFA
MIMIC-III: Medical Information Mart for Intensive Care
SBP: Systolic blood pressure
DBP: Diastolic blood pressure
MAP: Mean arterial pressure
SPO_2_: Pulse oxygen saturation
WBC: White blood cell count
RBC: Red blood count
PLT: Platelet count
BUN: Blood urea nitrogen
TBIL: Total bilirubin
MCHC: Mean corpuscular hemoglobin concentration
ALP: Alkaline phosphatase
ALT: Alanine aminotransferase
PO_2_: Oxygen partial pressure
PCO_2_: Partial pressure of carbon dioxide
SOFA: Sequential organ failure assessment
ICU: Intensive care unit
RRT: Renal replacement therapy
