# Supplementary material for: One-year mortality prediction for patients with sepsis: a nomogram integrating lactic dehydrogenase and clinical characteristics
Source: BMC Infect Dis. 2023 Oct 9;23:668. doi: 10.1186/s12879-023-08636-8 (PMC10561401; doi:10.1186/s12879-023-08636-8)
Supplement: Supplementary file 1 — Additional file 1: Supplemental Table 1. Sensitivity analysis of missing data before and after interpolation. Supplemental Table 2. The characteristics of patients in training set and testing set. Supplemental Table 3. The screening of predictors. [file 12879_2023_8636_MOESM1_ESM.docx]

**Supplemental Table 1. Sensitivity analysis of missing data before and after interpolation**

| **Variables** | **After the interpolation (n=1983)** | **Before the interpolation (n=1983)** | **Statistics** | ***P*** |
| --- | --- | --- | --- | --- |
| Marital status, n (%) |  |  | χ^2^=0.093 | 0.760 |
| Married | 970 (48.92) | 919 (49.41) |  |  |
| Not married | 1013 (51.08) | 941 (50.59) |  |  |
| Ethnicity, n (%) |  |  | χ^2^=1.755 | 0.625 |
| Asian | 56 (2.82) | 56 (3.09) |  |  |
| Black | 193 (9.73) | 193 (10.66) |  |  |
| Hispanic | 99 (4.99) | 99 (5.47) |  |  |
| White | 1635 (82.45) | 1463 (80.78) |  |  |
| Respiratory rate, M (Q_1_, Q_3_) | 21.00 (17.00, 25.00) | 21.00 (17.00, 25.00) | Z=-0.053 | 0.958 |
| Temperature, Mean ± SD | 36.73 ± 2.95 | 36.73 ± 2.96 | t=0.01 | 0.994 |
| Heart rate, Mean ± SD | 97.63 ± 21.71 | 97.61 ± 21.75 | t=0.02 | 0.982 |
| SBP, Mean ± SD | 115.17 ± 25.46 | 115.16 ± 25.50 | t=0.01 | 0.991 |
| DBP, M (Q_1_, Q_3_) | 60.00 (49.00, 72.00) | 60.00 (49.00, 72.00) | Z=0.047 | 0.962 |
| MAP, Mean ± SD | 76.42 ± 19.07 | 76.40 ± 19.10 | t=0.04 | 0.970 |
| SPO2, Mean ± SD | 95.82 ± 7.32 | 95.81 ± 7.34 | t=0.01 | 0.993 |
| pH, Mean ± SD | 7.34 ± 0.12 | 7.34 ± 0.12 | t=0.18 | 0.859 |
| Lactate, M (Q_1_, Q_3_) | 2.20 (1.50, 3.70) | 2.20 (1.50, 3.70) | Z=0.019 | 0.985 |
| Albumin, Mean ± SD | 2.90 ± 0.65 | 2.90 ± 0.66 | t=0.00 | 0.999 |
| TBIL, M (Q_1_, Q_3_) | 0.70 (0.40, 1.50) | 0.70 (0.40, 1.50) | Z=-0.060 | 0.952 |
| ALP, M (Q_1_, Q_3_) | 97.00 (69.00, 153.00) | 97.00 (69.00, 153.00) | Z=-0.037 | 0.970 |
| ALT, M (Q_1_, Q_3_) | 30.00 (17.00, 62.00) | 30.00 (17.00, 62.00) | Z=-0.014 | 0.989 |
| PO2, M (Q_1_, Q_3_) | 98.00 (69.00, 173.20) | 94.00 (66.00, 175.00) | Z=-1.052 | 0.293 |
| PCO2, M (Q_1_, Q_3_) | 39.00 (33.00, 47.00) | 39.00 (33.00, 47.00) | Z=-0.550 | 0.582 |

SBP, systolic blood pressure; DBP, diastolic blood pressure; MAP, mean arterial pressure; SPO_2_, pulse oxygen saturation; TBIL, total bilirubin; ALP, alkaline phosphatase; ALT, alanine aminotransferase; PO_2_, oxygen partial pressure PCO_2_, partial pressure of carbon dioxide.

**Supplemental Table 2. The characteristics of patients in training set and testing set**

| **Variables** | **Total (n=1983)** | **Testing set (n=595)** | **Training set (n=1388)** | **Statistics** | ***P*** |
| --- | --- | --- | --- | --- | --- |
| Age, years, Mean ± SD | 67.79 ± 15.31 | 67.25 ± 14.94 | 68.02 ± 15.47 | t=-1.03 | 0.303 |
| Gender, n (%) |  |  |  | χ^2^=1.608 | 0.205 |
| Female | 819 (41.30) | 233 (39.16) | 586 (42.22) |  |  |
| Male | 1164 (58.70) | 362 (60.84) | 802 (57.78) |  |  |
| Marital status, n (%) |  |  |  | χ^2^=1.152 | 0.283 |
| Married | 970 (48.92) | 302 (50.76) | 668 (48.13) |  |  |
| Not married | 1013 (51.08) | 293 (49.24) | 720 (51.87) |  |  |
| Ethnicity, n (%) |  |  |  | χ^2^=4.758 | 0.190 |
| Asian | 56 (2.82) | 13 (2.18) | 43 (3.10) |  |  |
| Black | 193 (9.73) | 51 (8.57) | 142 (10.23) |  |  |
| Hispanic | 99 (4.99) | 24 (4.03) | 75 (5.40) |  |  |
| White | 1635 (82.45) | 507 (85.21) | 1128 (81.27) |  |  |
| ICU type, n (%) |  |  |  | χ^2^=5.988 | 0.200 |
| CCU | 209 (10.54) | 69 (11.60) | 140 (10.09) |  |  |
| CSRU | 71 (3.58) | 15 (2.52) | 56 (4.03) |  |  |
| MICU | 1322 (66.67) | 385 (64.71) | 937 (67.51) |  |  |
| SICU | 260 (13.11) | 84 (14.12) | 176 (12.68) |  |  |
| TSICU | 121 (6.10) | 42 (7.06) | 79 (5.69) |  |  |
| Length of stay, days, M (Q_1_, Q_3_) | 5.82 (2.86, 13.06) | 6.03 (2.97, 13.63) | 5.67 (2.81, 12.73) | Z=1.286 | 0.198 |
| Respiratory rate, times/min, Mean ± SD | 21.00 (17.00, 25.00) | 21.00 (17.00, 25.00) | 21.00 (17.00, 25.00) | Z=1.312 | 0.189 |
| Temperature, ℃, Mean ± SD | 36.73 ± 2.95 | 36.75 ± 3.80 | 36.72 ± 2.50 | t=0.19 | 0.846 |
| Heart rate, times/min, Mean ± SD | 97.63 ± 21.71 | 98.26 ± 21.54 | 97.36 ± 21.79 | t=0.85 | 0.397 |
| SBP, mmHg, Mean ± SD | 115.17 ± 25.46 | 115.46 ± 25.64 | 115.05 ± 25.39 | t=0.33 | 0.738 |
| DBP, mmHg, Mean ± SD | 60.00 (49.00, 72.00) | 60.00 (50.00, 72.00) | 60.00 (49.00, 71.00) | Z=0.170 | 0.865 |
| MAP, mmHg, Mean ± SD | 76.42 ± 19.07 | 76.57 ± 18.75 | 76.36 ± 19.21 | t=0.22 | 0.826 |
| SPO_2_, Mean ± SD | 95.82 ± 7.32 | 95.80 ± 7.26 | 95.82 ± 7.35 | t=-0.07 | 0.946 |
| WBC, K/uL, M (Q_1_, Q_3_) | 11.90 (7.70, 17.50) | 11.70 (7.70, 17.70) | 11.90 (7.70, 17.40) | Z=-0.333 | 0.739 |
| RBC, m/uL, Mean ± SD | 3.75 ± 0.77 | 3.75 ± 0.78 | 3.75 ± 0.77 | t=-0.04 | 0.966 |
| Sodium, mEq/L, Mean ± SD | 137.47 ± 6.43 | 137.21 ± 6.14 | 137.59 ± 6.55 | t=-1.20 | 0.230 |
| Potassium, mEq/L, Mean ± SD | 4.42 ± 0.98 | 4.41 ± 0.94 | 4.43 ± 1.00 | t=-0.32 | 0.749 |
| Phosphate, mg/dL, M (Q_1_, Q_3_) | 3.60 (2.80, 4.60) | 3.50 (2.70, 4.40) | 3.60 (2.80, 4.60) | Z=-1.300 | 0.193 |
| Calcium mg/dL, Mean ± SD | 8.20 ± 1.06 | 8.18 ± 1.06 | 8.21 ± 1.06 | t=-0.64 | 0.525 |
| PLT, K/uL, M (Q_1_, Q_3_) | 213.00 (134.00, 303.00) | 214.00 (140.00, 305.00) | 210.00 (133.00, 303.00) | Z=0.714 | 0.475 |
| pH, Mean ± SD | 7.34 ± 0.12 | 7.34 ± 0.12 | 7.34 ± 0.12 | t=0.67 | 0.505 |
| Lactate, mmol/L, M (Q_1_, Q_3_) | 2.20 (1.50, 3.70) | 2.10 (1.50, 3.60) | 2.30 (1.50, 3.80) | Z=-1.699 | 0.089 |
| Magnesium, mg/dL, Mean ± SD | 1.93 ± 0.49 | 1.91 ± 0.46 | 1.94 ± 0.51 | t=-1.39 | 0.166 |
| Glucose, mg/dL, M (Q_1_, Q_3_) | 132.00 (106.00, 178.00) | 133.00 (105.00, 178.00) | 132.00 (106.00, 177.00) | Z=0.022 | 0.982 |
| Creatinine, mg/dL, M (Q_1_, Q_3_) | 1.50 (1.00, 2.50) | 1.40 (1.00, 2.50) | 1.50 (1.00, 2.50) | Z=-0.651 | 0.515 |
| BUN, mg/dL, M (Q_1_, Q_3_) | 31.00 (20.00, 50.00) | 29.00 (19.00, 48.00) | 31.50 (20.00, 51.00) | Z=-1.328 | 0.184 |
| Bicarbonate, mEq/L, Mean ± SD | 22.52 ± 5.56 | 22.43 ± 5.57 | 22.56 ± 5.56 | t=-0.48 | 0.629 |
| Albumin, Mean ± SD | 2.90 ± 0.65 | 2.89 ± 0.66 | 2.90 ± 0.65 | t=-0.44 | 0.657 |
| TBIL, mg/dL, M (Q_1_, Q_3_) | 0.70 (0.40, 1.50) | 0.70 (0.40, 1.60) | 0.70 (0.40, 1.50) | Z=0.652 | 0.514 |
| Hematocrit, %, Mean ± SD | 34.00 ± 6.45 | 34.02 ± 6.32 | 33.99 ± 6.51 | t=0.10 | 0.918 |
| Hemoglobin, g/dL, Mean ± SD | 11.27 ± 2.21 | 11.31 ± 2.20 | 11.25 ± 2.21 | t=0.49 | 0.627 |
| MCHC, Mean ± SD | 33.16 ± 1.65 | 33.26 ± 1.60 | 33.12 ± 1.66 | t=1.77 | 0.077 |
| ALP, M (Q_1_, Q_3_) | 97.00 (69.00, 153.00) | 93.00 (67.00, 150.00) | 99.00 (70.00, 155.00) | Z=-1.287 | 0.198 |
| ALT, U/L, M (Q_1_, Q_3_) | 30.00 (17.00, 62.00) | 30.00 (17.00, 60.00) | 30.00 (17.00, 64.00) | Z=-0.626 | 0.531 |
| AST, U/L, M (Q_1_, Q_3_) | 43.00 (25.00, 99.00) | 44.00 (25.00, 97.00) | 43.00 (25.00, 100.00) | Z=-0.326 | 0.745 |
| LDH, U/L, M (Q_1_, Q_3_) | 283.00 (209.00, 417.00) | 287.00 (204.00, 406.00) | 281.00 (213.00, 426.50) | Z=-1.194 | 0.232 |
| CK, U/L, M (Q_1_, Q_3_) | 113.00 (48.00, 292.00) | 116.00 (50.00, 303.00) | 113.00 (47.00, 285.00) | Z=0.570 | 0.569 |
| CK-MB, U/L, M (Q_1_, Q_3_) | 4.00 (3.00, 8.00) | 4.00 (3.00, 8.00) | 4.00 (3.00, 8.00) | Z=0.271 | 0.786 |
| PO_2_, M (Q_1_, Q_3_) | 98.00 (69.00, 173.20) | 98.00 (69.00, 162.00) | 98.00 (68.00, 178.20) | Z=-0.188 | 0.851 |
| PCO_2_, M (Q_1_, Q_3_) | 39.00 (33.00, 47.00) | 39.00 (32.00, 47.00) | 39.00 (33.00, 47.00) | Z=-1.102 | 0.271 |
| Congestive heart failure, n (%) |  |  |  | χ^2^=2.579 | 0.108 |
| No | 1062 (53.56) | 335 (56.30) | 727 (52.38) |  |  |
| Yes | 921 (46.44) | 260 (43.70) | 661 (47.62) |  |  |
| Malignant tumor, n (%) |  |  |  | χ^2^=0.007 | 0.931 |
| No | 1459 (73.58) | 437 (73.45) | 1022 (73.63) |  |  |
| Yes | 524 (26.42) | 158 (26.55) | 366 (26.37) |  |  |
| Renal failure, n (%) |  |  |  | χ^2^=0.338 | 0.561 |
| No | 572 (28.85) | 177 (29.75) | 395 (28.46) |  |  |
| Yes | 1411 (71.15) | 418 (70.25) | 993 (71.54) |  |  |
| Atrial fibrillation, n (%) |  |  |  | χ^2^=0.461 | 0.497 |
| No | 1147 (57.84) | 351 (58.99) | 796 (57.35) |  |  |
| Yes | 836 (42.16) | 244 (41.01) | 592 (42.65) |  |  |
| Respiratory failure, n (%) |  |  |  | χ^2^=0.015 | 0.901 |
| No | 869 (43.82) | 262 (44.03) | 607 (43.73) |  |  |
| Yes | 1114 (56.18) | 333 (55.97) | 781 (56.27) |  |  |
| Septic shock, n (%) |  |  |  | χ^2^=0.811 | 0.368 |
| No | 897 (45.23) | 260 (43.70) | 637 (45.89) |  |  |
| Yes | 1086 (54.77) | 335 (56.30) | 751 (54.11) |  |  |
| Elixhauser score, M (Q_1_, Q_3_) | 24.00 (14.00, 34.00) | 24.00 (15.00, 34.00) | 24.00 (14.00, 34.00) | Z=1.041 | 0.298 |
| SOFA total score, M (Q_1_, Q_3_) | 7.00 (5.00, 10.00) | 7.00 (5.00, 10.00) | 7.00 (5.00, 10.00) | Z=-0.133 | 0.894 |
| SAPSII, Mean ± SD | 47.44 ± 15.37 | 47.12 ± 15.40 | 47.58 ± 15.36 | t=-0.60 | 0.547 |
| RRT, n (%) |  |  |  | χ^2^=0.017 | 0.896 |
| No | 1777 (89.61) | 534 (89.75) | 1243 (89.55) |  |  |
| Yes | 206 (10.39) | 61 (10.25) | 145 (10.45) |  |  |
| Ventilation, n (%) |  |  |  | χ^2^=0.006 | 0.936 |
| No | 654 (32.98) | 197 (33.11) | 457 (32.93) |  |  |
| Yes | 1329 (67.02) | 398 (66.89) | 931 (67.07) |  |  |
| Vasopressor, n (%) |  |  |  | χ^2^=0.289 | 0.591 |
| No | 1479 (74.58) | 439 (73.78) | 1040 (74.93) |  |  |
| Yes | 504 (25.42) | 156 (26.22) | 348 (25.07) |  |  |
| Follow-up time, days, M (Q_1_, Q_3_) | 143.19 (15.45, 365.00) | 163.09 (16.36, 365.00) | 137.96 (15.05, 365.00) | Z=1.012 | 0.311 |
| One-year survival status, n (%) |  |  |  | χ^2^=0.782 | 0.376 |
| Survival | 870 (43.87) | 270 (45.38) | 600 (43.23) |  |  |
| Death | 1113 (56.13) | 325 (54.62) | 788 (56.77) |  |  |

ICU, intensive care unit; CCU, coronary care unit; CSRU, cardiac surgery recovery unit; MICU, medical intensive care unit; SICU, surgical intensive care unit; TSICU, trauma/surgical intensive care unit; SBP, systolic blood pressure; DBP, diastolic blood pressure; MAP, mean arterial pressure; SPO_2_, pulse oxygen saturation; WBC, white blood cell count; RBC, red blood count; PLT, platelet count; BUN, blood urea nitrogen; TBIL, total bilirubin; MCHC, mean corpuscular hemoglobin concentration; ALP, alkaline phosphatase; ALT, alanine aminotransferase; CK, creatine kinase; CK-MB, creatine kinase isoenzyme; AST, aspartic transoxygenase; LDH, lactic dehydrogenase; PO_2_, oxygen partial pressure; PCO_2,_ partial pressure of carbon dioxide; SOFA, sequential organ failure assessment; SAPSII, simplified acute physiology score II; RRT, renal replacement therapy.

**Supplemental Table 3. The screening of predictors**

| Variables | Univariate logistic regression | *P* |
| --- | --- | --- |
|  | OR (95%CI) |  |
| Age | 1.59 (1.42-1.78) | <0.001 |
| Gender |  |  |
| Female | Ref |  |
| Male | 1.25 (1.01-1.55) | 0.040 |
| Marital status |  |  |
| Married | Ref |  |
| Not married | 1.08 (0.87-1.33) | 0.499 |
| Ethnicity |  |  |
| Asian | Ref |  |
| Black | 1.81 (0.89-3.68) | 0.099 |
| Hispanic | 2.02 (0.93-4.38) | 0.074 |
| White | 2.67 (1.41-5.06) | 0.003 |
| ICU type |  |  |
| CCU | Ref |  |
| CSRU | 1.29 (0.67-2.48) | 0.453 |
| MICU | 0.75 (0.52-1.08) | 0.117 |
| SICU | 0.80 (0.51-1.26) | 0.339 |
| TSICU | 0.89 (0.51-1.57) | 0.699 |
| Length of stay | 1.02 (0.92-1.14) | 0.666 |
| Respiratory rate | 0.99 (0.89-1.10) | 0.881 |
| Temperature | 0.85 (0.71-1.01) | 0.062 |
| Heart rate | 0.89 (0.80-0.99) | 0.037 |
| SBP | 0.96 (0.87-1.07) | 0.478 |
| DBP | 0.83 (0.75-0.93) | <0.001 |
| MAP | 0.93 (0.84-1.03) | 0.180 |
| SPO2 | 0.96 (0.86-1.07) | 0.479 |
| WBC | 0.98 (0.88-1.08) | 0.650 |
| RBC | 0.75 (0.67-0.84) | <0.001 |
| Sodium | 0.98 (0.89-1.09) | 0.772 |
| Potassium | 1.38 (1.23-1.54) | <0.001 |
| Phosphate | 1.37 (1.22-1.54) | <0.001 |
| Calcium | 1.25 (1.12-1.39) | <0.001 |
| PLT | 0.99 (0.89-1.10) | 0.812 |
| pH | 1.07 (0.97-1.19) | 0.192 |
| Lactate | 1.11 (1.00-1.24) | 0.055 |
| Magnesium | 1.31 (1.17-1.46) | <0.001 |
| Glucose | 0.88 (0.78-0.98) | 0.017 |
| Creatinine | 1.09 (0.98-1.22) | 0.106 |
| BUN | 1.36 (1.21-1.54) | <0.001 |
| Bicarbonate | 1.09 (0.98-1.22) | 0.097 |
| Albumin | 0.83 (0.75-0.93) | <0.001 |
| TBIL | 1.19 (1.05-1.35) | 0.006 |
| Hematocrit | 0.77 (0.69-0.86) | <0.001 |
| Hemoglobin | 0.73 (0.65-0.82) | <0.001 |
| MCHC | 0.82 (0.73-0.91) | <0.001 |
| ALP | 1.45 (1.21-1.72) | <0.001 |
| ALT | 1.07 (0.95-1.21) | 0.268 |
| AST | 1.06 (0.94-1.20) | 0.332 |
| LDH | 1.28 (1.09-1.49) | 0.002 |
| CK | 0.94 (0.82-1.08) | 0.404 |
| CK-MB | 1.04 (0.93-1.16) | 0.508 |
| PO2 | 1.01 (0.91-1.12) | 0.886 |
| PCO2 | 1.02 (0.92-1.14) | 0.659 |
| Congestive heart failure |  |  |
| No | Ref |  |
| Yes | 1.26 (1.02-1.56) | 0.032 |
| Malignant tumor |  |  |
| No | Ref |  |
| Yes | 1.57 (1.23-2.01) | <0.001 |
| Atrial fibrillation |  |  |
| No | Ref |  |
| Yes | 1.51 (1.21-1.87) | <0.001 |
| Respiratory failure |  |  |
| No | Ref |  |
| Yes | 1.64 (1.33-2.04) | <0.001 |
| Septic shock |  |  |
| No | Ref |  |
| Yes | 1.13 (0.92-1.40) | 0.247 |
| Elixhauser score | 1.83 (1.62-2.06) | <0.001 |
| SOFA total score | 1.35 (1.21-1.51) | <0.001 |
| SAPS II | 1.96 (1.73-2.23) | <0.001 |
| RRT |  |  |
| No | Ref |  |
| Yes | 1.67 (1.16-2.41) | 0.006 |
| Ventilation |  |  |
| No | Ref |  |
| Yes | 1.48 (1.18-1.85) | <0.001 |
| Vasopressor |  |  |
| No | Ref |  |
| Yes | 2.18 (1.68-2.82) | <0.001 |

CK, creatine kinase; CK-MB, creatine kinase isoenzyme; AST, aspartic transoxygenase; LDH, lactic dehydrogenase; OR, odds ratio; CI, confidence interval;

Model 1, crude model; Model 2, adjusted age, gender, ethnicity, potassium, calcium, albumin, hemoglobin, alkaline phosphatase, vasopressor, Elixhauser score and respiratory failure.
